# An Introductory Lecture

**Published:** 1854-02

**Authors:** 


*An Introductory Lecture.* By Benj. R. Palmer, M. D., Professor of Anatomy in the Medical Department of the University of Louisville.

This is a well written, and—what is better—a thoughtful production. Dr. Palmer urges the importance of *all* knowledge, in contradistinction to the notions of those who, calling themselves *par excellence* "practical men," can see no utility in the researches of microscopy, or in the attainment of isolated facts which have no direct bearing on therapeutic art. His argument in this connection is forcibly put, and illustrated.

We cannot avoid the conclusion, that the task of writing an "introductory," which Dr. Palmer speaks of in the opening page as so irksome and disagreeable, must have grown lighter in the fulfillment. No man writes well who writes unwillingly, and hence we draw the conclusion, that our lecturer must have warmed with his subject as he progressed; writing at least *currente calamo*, and with a hearty appreciation of the importance and interest of his theme. H.

